# Supplementary material for: Concept neurons in the human medial temporal lobe flexibly represent abstract relations between concepts
Source: Nat Commun. 2021 Oct 25;12:6164. doi: 10.1038/s41467-021-26327-3 (PMC8545952; doi:10.1038/s41467-021-26327-3)
Supplement: Supplementary file 1 — Supplementary Information [file 41467_2021_26327_MOESM1_ESM.pdf]

Supplementary Information

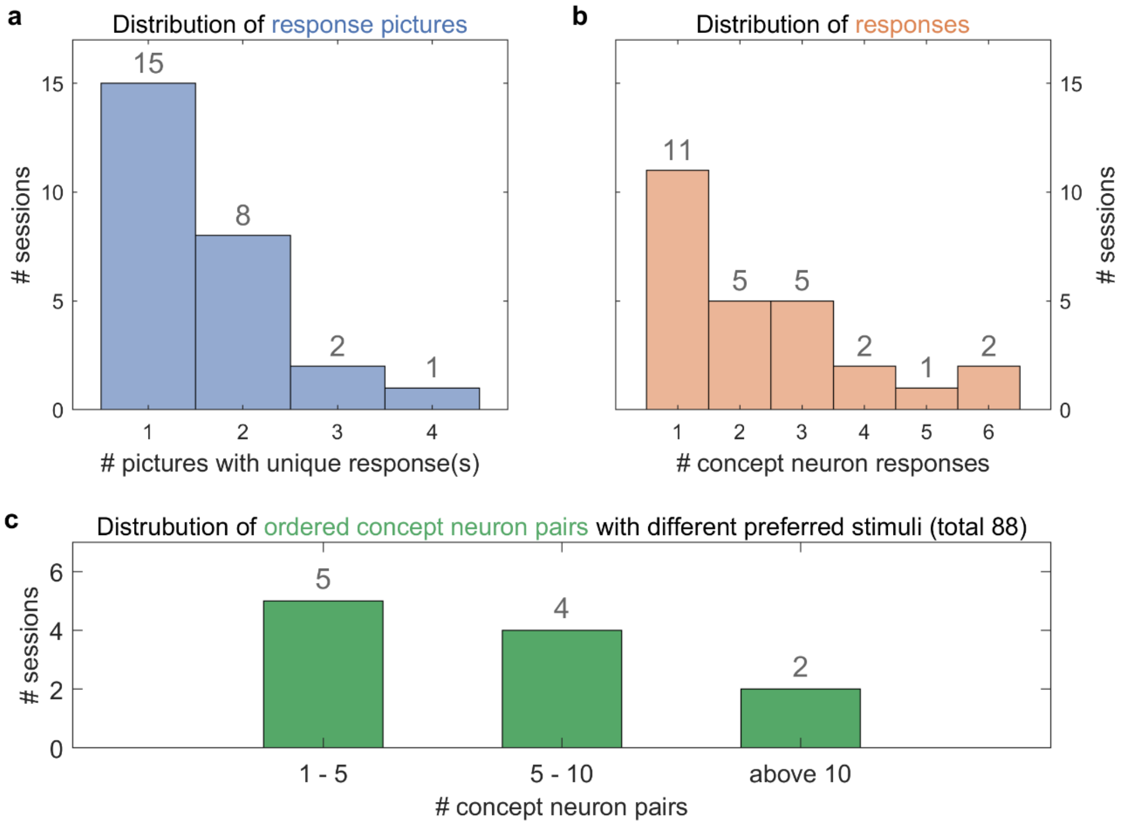

Supplementary Figure 1. a. Distribution of the number of response-eliciting pictures with at least one unique concept-neuron response across sessions. In 11 sessions, 2 or more pictures elicited at least one unique concept-neuron response. **b.** Distribution of concept-neuron responses across sessions (including cases where multiple concept neurons respond to the same picture included). In 15 sessions there were 2 or more concept neuron responses. **c.** Distribution of ordered concept-neuron pairs with different preferred stimuli.

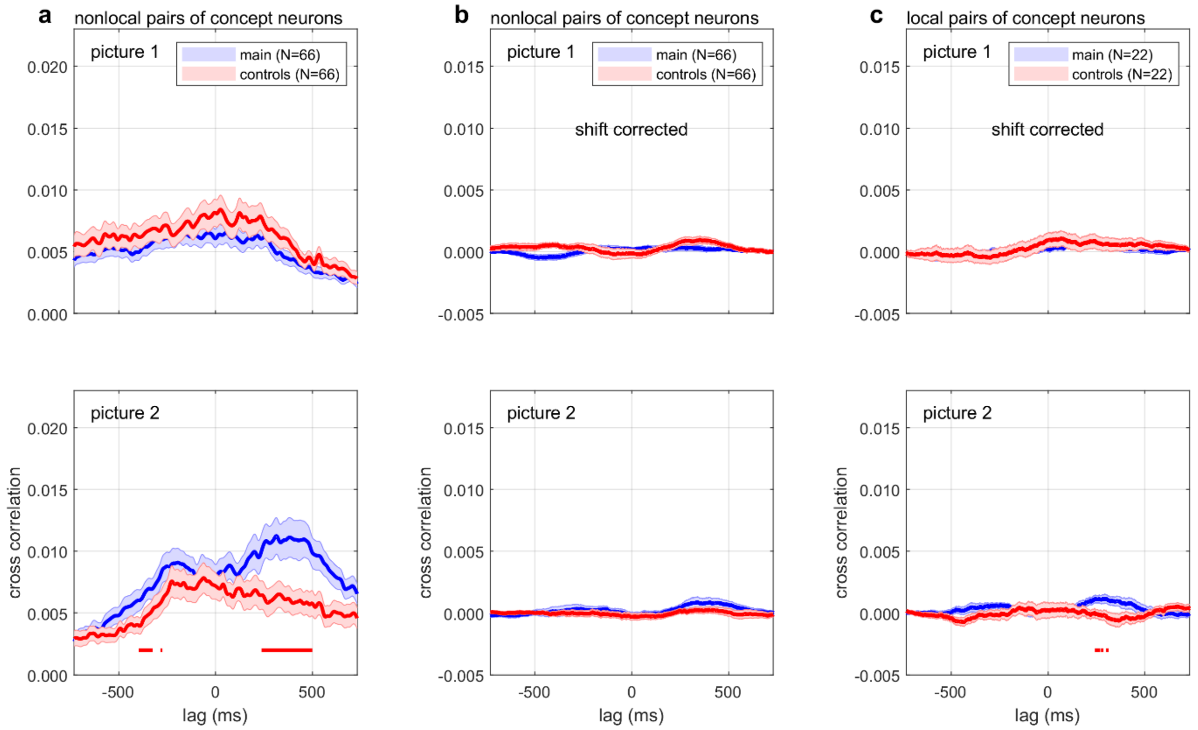

Supplementary Figure 2. Cross-correlograms for non-local pairs (a) and after subtraction of shift-predictors (b and c). **a.** Population plots of trial-by-trial cross-correlograms between all 66 non-local pairs of concept neurons for trials in which both (non-identical) preferred concepts were depicted in either the main experiment (blue) or the control conditions (red). **b.** Same as a but with shift correction obtained by subtracting cross-correlograms from non-simultaneous pairwise activity of consecutive trials (shift predictor). **c.** Same as b but calculated for all 22 local pairs of concept neurons from the same wire bundle. Red horizontal lines indicate significant differences (p<0.05) between the main comparison condition and controls as quantified by a two-sided cluster permutation test (a-c). Data are presented as mean values +/- SEM (a-c) with solid lines and shaded areas, respectively.

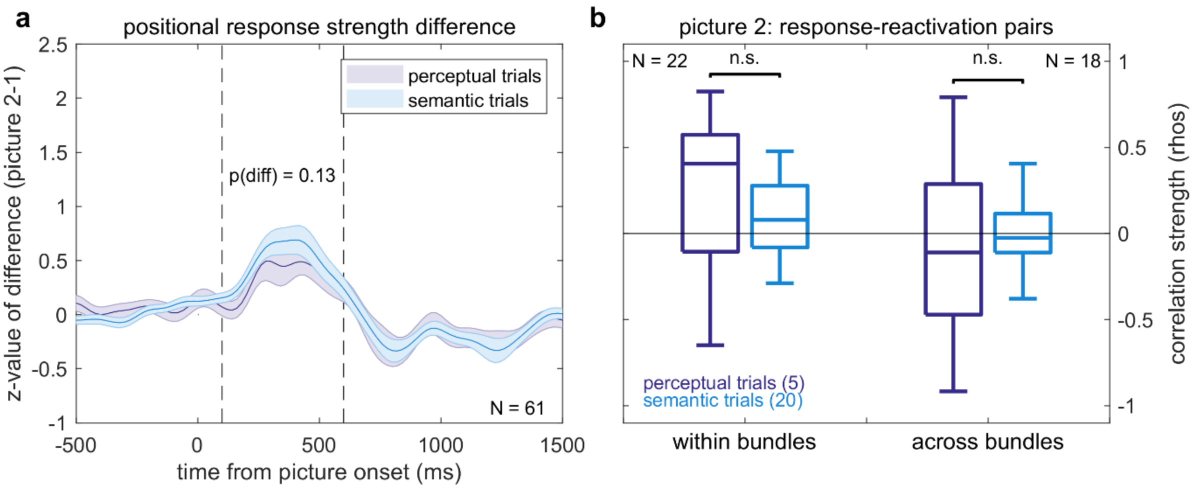

Supplementary Figure 3. Positional response strength differences and pairwise correlations did not differ significantly between perceptual and semantic trials. **a.** Normalized positional difference of concept-neuron responses (N=61) to the preferred concept in second minus first picture position for perceptual (dark blue) versus semantic (blue) trials. Data are presented as mean values +/- SEM (solid lines and shaded areas). Positional differences were slightly more pronounced in semantic trials without reaching significance (two-sided Wilcoxon signed-rank test, 100-600 ms, p=0.13). **b.** Effect sizes of Spearman correlations of firing rates for pairs of concept neurons in semantic (blue) versus perceptual (dark blue) trials are visualized as boxplots (Q1, median, Q3; whisker: points within +/- 1.5 IQR). Activity from each pair of neurons n2 and n1 was obtained during the second picture response (n2: 0-1000 ms) or reactivation window (n1: 500-1300 ms) and from trials in which both (non-identical) preferred concepts were shown (first that of n1, then that of n2). Effect sizes of pairwise correlations were not significantly different in semantic versus perceptual trials, neither within nor across bundles of the same hemisphere (two-sided Mann-Whitney U test within: p=0.09, across: p=0.32)

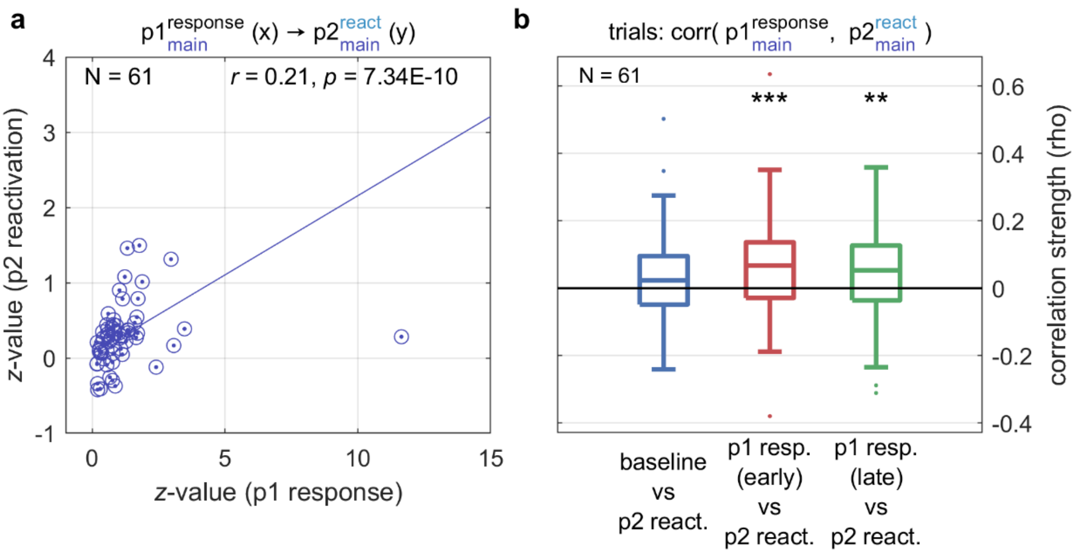

Supplementary Figure 4. Response strengths to preferred concepts shown first predict reactivation strengths both on a population (a) and on a trial level (b), pointing to potential differences in excitability or intrinsic plasticity due to previous activation. **a.** Scatter plot of mean normalized population activity of concept neurons between preferred picture responses ($p1^{response}$: picture 1 response, 0-1000 ms) and reactivations ($p2^{react}$: picture 2 reactivation, 500-1300 ms) from reactivation trials of the main experiment as denoted by the subscript**.** Results of a linear regression model ("fitlm" in MATLAB 2016b, top right corner) with outlier correction (Iteratively Reweighted Least Squares) are visualized by regression lines. Population responses to first preferred pictures significantly correlate with population reactivation strengths (r=0.21, $p<10^{-9}$, uncorrected). **b.** Distributions of trial-wise correlation effect sizes between either baseline (-400-100 ms, left), early (100-600 ms, middle) or late (600-1100 ms, right) response activity to the preferred picture in first position (p1 resp.) and reactivations (p2 react.: 500-1300 ms) are visualized as boxplots (Q1, median, Q3; whisker: points within +/- 1.5 IQR). Two-sided Wilcoxon signed-rank tests against zero revealed that both early (z=3.42, $p=6.20\times10^{-4}$) and late responses (z=2.90, $p=3.75\times10^{-3}$), but not baseline activities (z=1.56, p=0.12) predict trial-wise fluctuations in reactivation strengths.

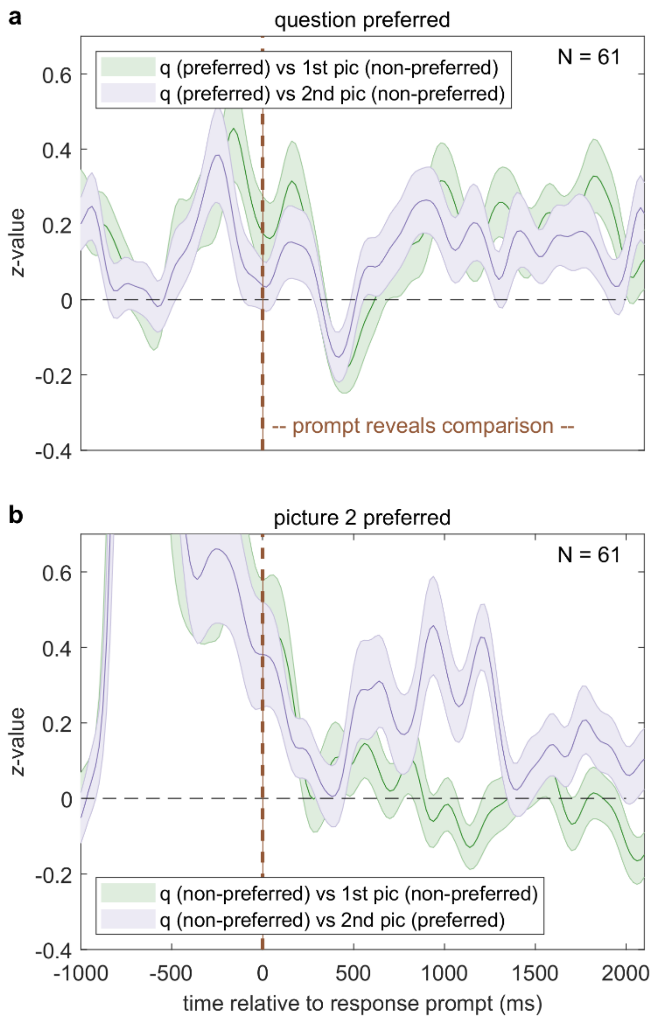

Supplementary Figure 5. Firing of concept neurons indicates whether attention is directed towards or away from the preferred concept. **a.** Averaged normalized firing rates of all concept neurons with standard errors (shaded areas) in the "question comparison" condition during the presentation of the response prompt at the end of each trial whenever the question contained the preferred concept. The response prompt asked for a comparison between the concept of the question (preferred) and either that of the first (non-preferred, green) or that of the second picture (non-preferred violet). Normalized activity did not differ significantly between these two instructions (p<0.01; two-sided cluster permutation test). In both cases the preferred concept of the question was relevant and normalized firing was increased apart from a short interruption upon presentation of the response prompt. **b.** Same as a but for trials in which the second picture contained the preferred concept. Concept neurons were reactivated only if the prompt referred to the preferred concept (violet) by asking for a comparison to the second picture. Following complete activity silence, reactivations began 500 ms after presentation of the response prompt (also see reactivation window). However, differences were not significant (p<0.01; two-sided cluster permutation test). Data are presented as mean values +/- SEM (a, b) with solid lines and shaded areas, respectively.

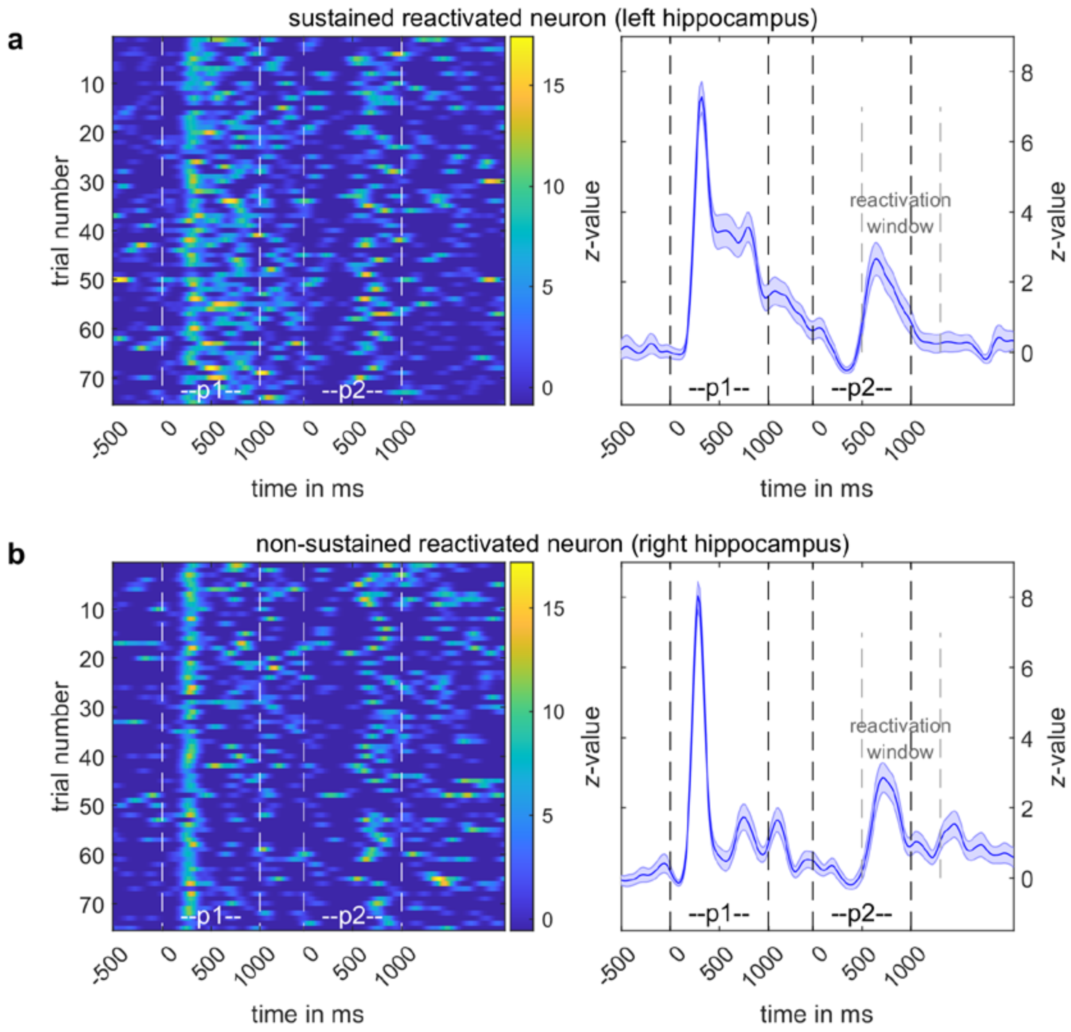

Supplementary Figure 6. Persistence of firing varies between concept neurons **a.** Left: Heat plot of *z*-values of a concept neuron in the left hippocampus across trials in which the preferred concept was depicted first (main comparison condition). Dashed white lines denote onsets and offsets of picture presentations (p1: picture 1, p2: picture 2). After the response to the first, preferred picture, firing was increased for almost the entire duration of the trial with the exception of a short period of activity silence during the presentation of the second, non-preferred pictures. Right: Corresponding averaged normalized firing rates. Solid lines and shaded areas depict means and standard errors, respectively. **b.** Same as a, but for a different concept neuron in the right hippocampus of the same patient, but from a different experimental session. Here, after a short response to the preferred picture in first position, activity was not sustained (if at all with brief rhythmic bouts of activity), yet still reactivated in the later phase of the presentation of the non-preferred pictures in second position after a longer period of activity silence.
