# Supplementary material for: Concept neurons in the human medial temporal lobe flexibly represent abstract relations between concepts
Source: Nat Commun. 2021 Oct 25;12:6164. doi: 10.1038/s41467-021-26327-3 (PMC8545952; doi:10.1038/s41467-021-26327-3)
Supplement: Supplementary file 3 — Reporting Summary [file 41467_2021_26327_MOESM3_ESM.pdf]

nature portfolio

Corresponding author(s): Florian Mormann

Last updated by author(s): 2021/09/20

Reporting Summary

Nature Portfolio wishes to improve the reproducibility of the work that we publish. This form provides structure for consistency and transparency in reporting. For further information on Nature Portfolio policies, see our Editorial Policies and the Editorial Policy Checklist.

Statistics

For all statistical analyses, confirm that the following items are present in the figure legend, table legend, main text, or Methods section.

n/a	Confirmed	
☐	☒	The exact sample size (*n*) for each experimental group/condition, given as a discrete number and unit of measurement
☐	☒	A statement on whether measurements were taken from distinct samples or whether the same sample was measured repeatedly
☐	☒	The statistical test(s) used AND whether they are one- or two-sided<br>*Only common tests should be described solely by name; describe more complex techniques in the Methods section.*
☐	☒	A description of all covariates tested
☐	☒	A description of any assumptions or corrections, such as tests of normality and adjustment for multiple comparisons
☐	☒	A full description of the statistical parameters including central tendency (e.g. means) or other basic estimates (e.g. regression coefficient) AND variation (e.g. standard deviation) or associated estimates of uncertainty (e.g. confidence intervals)
☐	☒	For null hypothesis testing, the test statistic (e.g. *F*, *t*, *r*) with confidence intervals, effect sizes, degrees of freedom and *P* value noted<br>*Give P values as exact values whenever suitable.*
☒	☐	For Bayesian analysis, information on the choice of priors and Markov chain Monte Carlo settings
☒	☐	For hierarchical and complex designs, identification of the appropriate level for tests and full reporting of outcomes
☐	☒	Estimates of effect sizes (e.g. Cohen's *d*, Pearson's *r*), indicating how they were calculated

Our web collection on statistics for biologists contains articles on many of the points above.

Software and code

Policy information about availability of computer code

Data collection	recordings: Neuralynx ATLAS system<br>spike-sorting software: Combinato, https://github.com/jniediek/combinato<br>stimulus delivery: Psychtoolbox3 (www.psythoolbox.org) with Octave (www.gnu.org/octave) on a Debian 8 operating system (www.debian.org)
Data analysis	MATLAB 2016b (functions ranksum, signrank, fitlm)<br>Code related to the main analyses of the manuscript is available at https://github.com/mabausch/ConceptNeuronRelations.git.

For manuscripts utilizing custom algorithms or software that are central to the research but not yet described in published literature, software must be made available to editors and reviewers. We strongly encourage code deposition in a community repository (e.g. GitHub). See the Nature Portfolio guidelines for submitting code & software for further information.

Data

Policy information about availability of data

All manuscripts must include a data availability statement. This statement should provide the following information, where applicable:

- Accession codes, unique identifiers, or web links for publicly available datasets
- A description of any restrictions on data availability
- For clinical datasets or third party data, please ensure that the statement adheres to our policy

All data supporting the findings of this study are publicly available at https://github.com/mabausch/ConceptNeuronRelations.git. Source data are provided with this paper.

Field-specific reporting

Please select the one below that is the best fit for your research. If you are not sure, read the appropriate sections before making your selection.

☐ Life sciences ☒ Behavioural & social sciences ☐ Ecological, evolutionary & environmental sciences

For a reference copy of the document with all sections, see nature.com/documents/nr-reporting-summary-flat.pdf

Behavioural & social sciences study design

All studies must disclose on these points even when the disclosure is negative.

Study description	quantitative (electrophysiological recordings during behavioural experiments)
Research sample	All patients that underwent treatment for pharmacologically intractable epilepsy partook in the experiment (11 right handed, 1 ambidextrous; 6 male; 22–65 years old). The sample is representative of patients with intractable epilepsy. The chosen study sample was constrained by the necessity to obtain single neuron recordings in humans.
Sampling strategy	The number of sessions necessary for a sufficient yield of concept neurons could be estimated based on previous reports (Quiroga et al. 2005) and our known yield of visually-selective neurons per session. 61 concept neurons could be detected from 26 experiments of 10 patients for statistical comparisons between different conditions and the total number of trials (N=300) resulted in reliable estimates of mean normalized activity for each condition.
Data collection	Electrode locations were planned exclusively based on clinical considerations. Each depth electrode contained a micro-wire bundle (AdTech, Racine, WI). The differential signal from the micro-wires was amplified using a Neuralynx ATLAS system (Bozeman, MT), filtered between 0.1 Hz and 9,000 Hz, and sampled at 32 kHz. Experiments were performed by each patient on a laptop computer while a researcher familiar with the research questions was present at a distance without knowledge of the conditions that were presented on screen.
Timing	07/24/2015 - 02/02/2017
Data exclusions	No data was excluded.
Non-participation	No dropouts.
Randomization	There was no allocation to experimental groups.

Reporting for specific materials, systems and methods

We require information from authors about some types of materials, experimental systems and methods used in many studies. Here, indicate whether each material, system or method listed is relevant to your study. If you are not sure if a list item applies to your research, read the appropriate section before selecting a response.

Materials & experimental systems

n/a	Involved in the study	
☒	☐	Antibodies
☒	☐	Eukaryotic cell lines
☒	☐	Palaeontology and archaeology
☒	☐	Animals and other organisms
☐	☒	Human research participants
☒	☐	Clinical data
☒	☐	Dual use research of concern

Methods

n/a	Involved in the study	
☒	☐	ChIP-seq
☒	☐	Flow cytometry
☒	☐	MRI-based neuroimaging

Human research participants

Policy information about studies involving human research participants

Population characteristics	12 patients with pharmacologically intractable epilepsy (11 right handed, 1 ambidextrous; 6 male; 22–65 years old)
Recruitment	All patients that underwent treatment for pharmacologically intractable epilepsy were asked to partake in the experiment and agreed. Therefore, there was no selection bias.
Ethics oversight	The study was approved by the Medical Institutional Review Board at the University of Bonn.

Note that full information on the approval of the study protocol must also be provided in the manuscript.
